# Genetic Diversity and Epidemiological Overlap of *Staphylococcus aureus* at the Animal–Food–Environment–Human Interface Within a One Health Framework

**DOI:** 10.1002/mbo3.70397

**Published:** 2026-09-03

**Authors:** Pinar Sagiroglu, Dursun Alp Gundog, Candan Gungor, Kursat Koskeroglu, Mustafa Altay Atalay, Adalet Dishan, Yeliz Ucar, Aytac Akcay, Huseyin Burak Disli, Harun Hizlisoy, Mukaddes Barel, Tekin Kececi, Nurhan Ertas Onmaz

**Affiliations:** ^1^ Department of Medical Microbiology, Faculty of Medicine Erciyes University Kayseri Türkiye; ^2^ Veterinary Faculty, Department of Food Hygiene and Technology Yozgat Bozok University Yozgat Türkiye; ^3^ Department of Veterinary Food Hygiene and Technology, Faculty of Veterinary Medicine Necmettin Erbakan University Ereğli Türkiye; ^4^ Department of Veterinary Public Health, Faculty of Veterinary Medicine Erciyes University Kayseri Türkiye; ^5^ Department of Biostatistics Ankara University, Veterinary Faculty Ankara Türkiye; ^6^ Eville and Jones, Century House Leeds UK

**Keywords:** CA‐MRSA, genetic diversity, LA‐MRSA, One Health, SCC*mec*, *spa* type

## Abstract

This study aimed to assess the genetic diversity and potential epidemiological overlap of *Staphylococcus aureus* using molecular (spa and SCCmec typing) and phenotypic characterization of 108 isolates obtained along the farm‐to‐fork continuum (dairy and meat chains) and 50 human clinical isolates. Forty‐four *spa* types, including 17 novel patterns, were identified, with t11284 and t127 predominating among animal‐related MRSA and clinical MRSA, respectively. Six SCCmec types (I–VI) were detected in the majority of isolates (85.2%), with SCCmec IVa prevalent in farm‐to‐fork isolates (67%) and SCCmec III dominant in clinical isolates (28%). Spa repeat‐based MST analysis revealed a heterogeneous distribution of isolates across clusters, with identical spa types detected in multiple source categories, indicating genetic relatedness rather than direct transmission events. Overall, 54.6% of isolates exhibited a multidrug‐resistant phenotype. Farm‐to‐fork isolates showed mainly β‐lactam resistance (≥ 85%), whereas clinical MRSA exhibited broader resistance profiles, including high fluoroquinolone resistance (≥ 92%). PVL was detected in 41 isolates (38%), predominantly in MRSA, and was associated with SCCmec IV/V and diverse spa types. Toxin genes (*tst*−1, *sea*, *seb*, and *sed*) were mainly confined to clinical MRSA, suggesting source‐associated distribution of virulence determinants. Biofilm formation was observed in 49 isolates (45.3%), more frequently among farm‐to‐fork isolates. Our study demonstrate marked genetic and phenotypic diversity of *S. aureus* across farm‐to‐fork and human clinical sources and suggest the presence of shared genetic lineages among isolates from different sources. The results support the importance of integrated One Health surveillance for monitoring antimicrobial‐resistant and virulent *S. aureus* populations across interconnected ecological compartments.

## Graphical abstract

Integrating farm‐to‐fork and clinical surveillance, this study reveals distinct SCCmec distributions alongside shared spa types across human, animal, and food interfaces. The results of genetic diversity and epidemiological overlap underscore the critical need for coordinated One Health surveillance to monitor multi‐sectoral Staphylococcus aureus transmission.

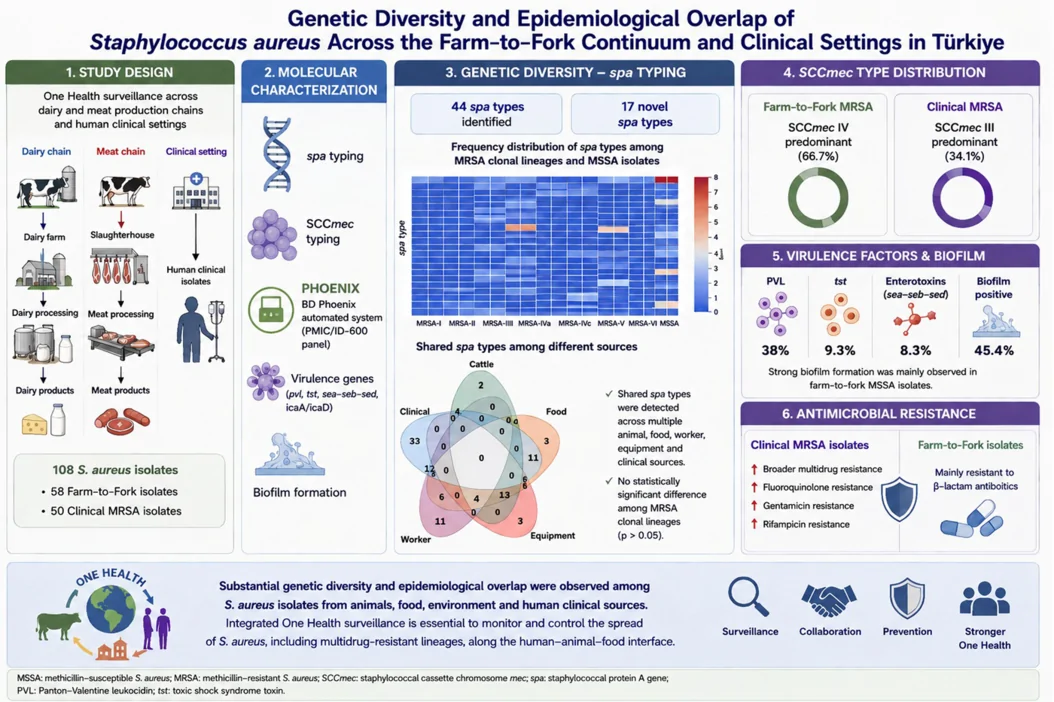

## 1 Introduction

Methicillin‐resistant *Staphylococcus aureus* (MRSA) is an emerging zoonotic pathogen and is classified as a Priority 2 (high) organism on the WHO (2017) global list of antibiotic‐resistant bacteria. Livestock can act as reservoirs of antibiotic resistance genes that may be shared with human‐associated MRSA (Boswihi et al. 2024; Liu et al. 2021; Tang et al. 2024). MRSA was initially described as hospital‐associated MRSA (HA‐MRSA) but has since spread to community‐associated (CA‐MRSA) and livestock‐associated (LA‐MRSA) settings (Khairullah et al. 2023).

MRSA remains a significant public health concern, with CA‐MRSA and HA‐MRSA causing a wide range of infections (Crippa et al. 2025; W. Zhang et al. 2024). CA‐MRSA frequently carries *pvl* (*luk*S/F‐PV), a potent cytotoxin that causes leukocyte destruction, tissue necrosis, and recurrent skin and soft tissue infections (Wójcik‐Bojek et al. 2022). However, its detection in HA‐MRSA has become increasingly common, blurring the epidemiological boundary between these lineages (Amin et al. 2020; Shohayeb et al. 2023).

MRSA is a versatile pathogen capable of causing painful skin infections as well as severe invasive disease in humans (Gehrke et al. 2023; Jones et al. 2021). Animals and their products (e.g., meat and milk) also serve as important reservoirs for LA‐MRSA in humans (Pauly et al. 2019). Notably, MRSA prevalence in animals has not declined, despite substantial reductions in antimicrobial use across most European countries between 2010 and 2017 (Crespo‐Piazuelo and Lawlor 2021).

The European Centre for Disease Prevention and Control (ECDC) reported increasing detection and wider geographical spread of LA‐MRSA in humans across the European Union between 2007 and 2013, highlighting its relevance within the One Health framework (Kinross et al. 2017). Characterizing the genetic diversity of *S. aureus* from different hosts and environments supports epidemiological investigations by clarifying patterns of distribution and genetic relatedness. Effective MRSA control requires coordinated interdisciplinary and intersectoral action within the One Health framework.

This study aimed to evaluate the genetic diversity, antimicrobial resistance profiles, virulence determinants, and potential epidemiological overlap of *S. aureus* isolates obtained from farm‐to‐fork and human clinical sources in Türkiye within a One Health framework. The objectives include: (i) genetic characterization of MRSA isolates from diverse habitats; (ii) detection of population‐level diversity; and (iii) investigation of relationships among isolates from different hosts.

## 2 Material and Methods

### 2.1 Isolates

In this study, 108 *S. aureus* isolates were analyzed, including 58 isolates obtained from a farm‐to‐fork surveillance project designed to evaluate the traceability and persistence of foodborne pathogens along the dairy and meat production continuum, and 50 clinical MRSA isolates from the culture collection of the Microbiology Department, Faculty of Medicine, Erciyes University, for comparative analysis of molecular and genetic characteristics between production‐chain and clinical populations. The detailed sampling framework, including selection criteria and characteristics of the sampled farms and production facilities, is provided in the Supplementary Material. All isolates were processed according to ISO 6888‐1, 6888‐1 (2021). Following primary isolation on Baird‐Parker agar, colonies with a typical black appearance surrounded by a clear zone were considered presumptive *S. aureus*, and up to five suspect colonies from each sample were further evaluated by catalase and coagulase tests. Presumptive isolates were initially identified by *nuc* gene‐targeted PCR, and species confirmation and antimicrobial susceptibility testing were subsequently performed using the BD Phoenix automated system (PMIC/ID‐600 panel), covering 24 antimicrobial agents: amikacin, amoxicillin/clavulanic acid, ampicillin, cefoxitin, ciprofloxacin, clindamycin, daptomycin, erythromycin, fosfomycin with glucose‐6‐phosphate, fusidic acid, gentamicin, levofloxacin, linezolid, moxifloxacin, nitrofurantoin, oxacillin, penicillin G, rifampin, streptomycin‐synergy, teicoplanin, tetracycline, trimethoprim/sulfamethoxazole, and vancomycin. AST results were interpreted according to EUCAST (2026) breakpoints (Table S1), β‐Lactamase‐producing MSSA isolates were classified as resistant to ampicillin; however, susceptibility to amoxicillin/clavulanic acid was retained because clavulanic acid inhibits β‐lactamase activity. Conversely, MRSA isolates were interpreted as resistant to both agents as part of the β‐lactam resistance phenotype associated with cefoxitin and/or oxacillin resistance. Methicillin resistance was determined using cefoxitin and oxacillin susceptibility testing and confirmed by *mecA* PCR. β‐lactamase production and MLSB/MSB phenotypes were interpreted using the Phoenix system based on the nitrocefin chromogenic reaction and erythromycin–clindamycin susceptibility profiles, respectively. MDR was defined as non‐susceptibility to at least one agent in three or more antimicrobial classes (Magiorakos et al. 2012).

### 2.2 Phenotypic Biofilm Formation Assay

Biofilm formation was assessed using the crystal violet microtiter plate assay described by Stepanović et al. (2000). *S. aureus* isolates were grown in sterile Trypticase Soy Broth (TSB) overnight, and cultures were diluted 1:100 in fresh TSB supplemented with 0.2% glucose. Diluted cultures were transferred to sterile round‐bottom 96‐well plates and incubated at 37°C for 18 h. Medium‐only wells served as negative controls to establish the cutoff value (ODc), and *S. aureus* ATCC 25923 was included as a positive control. After incubation, wells were gently washed with water to remove non‐adherent cells, and biofilms were stained with 0.1% crystal violet. Excess dye was removed by repeated washing, and plates were dried at room temperature. Bound crystal violet was solubilized with 33% (v/v) glacial acetic acid, and the OD of each well was measured at 570 nm using a ELISA reader (Thermo‐Scientific, Waltham, USA). All isolates were evaluated in triplicate across three independent experiments. According to their OD value, the tested isolates were classified as follows: OD ≤ ODc (non‐biofilm), ODc < OD ≤ 2 × ODc (weak), 2 × ODc < OD ≤ 4 × ODc (moderate), and OD > 4 × ODc (strong).

### 2.3 Molecular Identification and Characterization

For molecular characterization, genomic DNA was extracted according to the manufacturer's instructions using InstaGene Matrix (Bio‐Rad, USA).

Primers targeting the *mecA* gene encoding methicillin resistance; the *tst* gene encoding toxic shock syndrome toxin‐1 (TSST‐1); enterotoxin genes (*sea–see*); biofilm‐associated adhesion genes (*icaA, icaD, fnbA*, and *fnbB*); and Panton–Valentine leukocidin genes (*lukS‐PV/lukF‐PV*) were adopted from Mehrotra et al. (2000), Ertas et al. (2010), Akyol et al. (2023), and Vittorakis et al. (2023), respectively. *SCCmec* typing (I‐VI) of *mecA*‐positive isolates was carried out in accordance with Zhang et al. (2005) and Bhowmik et al. (2019).

All PCR assays were performed in a final volume of 25 µL containing 12.5 µL of 2× DreamTaq Green PCR Master Mix (Thermo Fisher Scientific, USA), 0.5 µL of each primer (25 pmol), 2 µL of template DNA, and *nuc*lease‐free water. Multiplex PCR assays consisted of four primer sets: Set A for *mecA* and *tst*; Set B for *sea–see*; and Sets C and D for *icaA/D and fnbA/B*. In addition, *lukS‐PV/lukF‐PV* and *SCCmec* typing were amplified in separate reactions.

PCR amplification consisted of an initial denaturation at 94°C for 3 min, followed by 30–35 cycles of denaturation at 95°C for 30 s, annealing for 30 s at primer‐specific temperatures, and extension at 72°C for 1 min, with a final extension at 72°C for 10 min. Amplified products were separated on 1.5% agarose gels by electrophoresis at 100 V for 60 min and visualized under UV transillumination using a gel documentation system (Biorad Gel Doc XR, USA). Primer sequences, amplicon sizes, and annealing temperatures are presented in Table 1. The following reference *S. aureus* strains were used as quality controls for PCR assays: ATCC 25923 (*mecA* − ; *nuc* + ; *pvl* + ; *icaA* + ; *icaD* + ; *fnbA* + ; *fnbB* + ), ATCC 43300 (*mecA* + ; *nuc* + ; *pvl* − ; *icaA* + ; *icaD* + ; *fnbA* + ; *fnbB* + ; SCCmec II), ATCC 51650 (*tst* + ), NCTC 10442 (SCCmec I), ATCC 33592 (*mecA* + ; SCCmec III), ATCC BAA‐1762 (*mecA* + ; SCCmec IV), ATCC BAA‐2094 (*mecA* + ; SCCmec V), NCTC 10652 (*nuc* + ; *mecA* − ; *sea* + ; *sed* + ), NCTC 10654 (*nuc* + ; *mecA* − ; *seb* + ), and NCTC 10655 (*nuc* + ; *mecA* − ; *sec* + ).

**Table 1:** Primer Sequences and Thermal Cycling Parameters for Molecular Identification and Typing of *S. aureus* Isolates.

Primer set	Target gene	Primer	Primer sequence (5′‐3′)	Product size	Cycling condition		References
					Annealing (°C/s)	Cycle	
Set A	*mecA*	*mecA*‐1	ACTGCTATCCACCCTCAAA	163	57°C/120	35	(Mehrotra et al. (2000))
		*mecA*‐2	CTGGTGAAGTTGTAATCTGG				
	*tst*	tst‐1	ACCCCTGTTCCCTTATCATC	326			
		tst‐2	TTTTCAGTATTTGTAACGCC				
Set B	*SA‐U*	SE‐F	TGTATGTATGGAGGTGTAAC	—	50°C/30	35	(Ertas et al. (2010))
	*SA‐A*	sea‐R	ATTAACCGAAGGTTCTGT	270			
	*SA‐B*	seb‐R	ATAGTGACGAGTTAGGTA	165			
	*SA‐C*	sec‐R	AAGTACATTTTGTAAGTTCC	69			
	*ENT‐C*	sec‐R	AATTGTGTTTCTTTTATTTTCATAA	102			
	*SA‐D*	sed‐R	TTCGGGAAAATCACCCTTAA	306			
	*SA‐E*	see‐R	GCCAAAGCTGTCTGAG	213			
Set C	*icaA*	icaA‐F	TCTCTTGCAGGAGCAATCAA	188	55°C/30	35	(Akyol et al. (2023))
		icaA‐R	TCAGGCACTAACATCCAGCA				
	*fnbB*	fnbB‐1	CGTTATTTGTAGTTGTTTGTGTT	118			
		fnbB‐2	TGGAATGGGACAAGAAAAAGAA				
Set D	*icaD*	icaD‐1	ATGGTCAAGCCCAGACAGAG	198			
		icaD‐2	CGTGTTTTCAACATTTAATGCAA				
	*fnbA*	fnbA‐1	ACTTGATTTTGTGTAGCCTTTTT	185			
		fnbA‐2	GAAGAAGCACCAAAAGCAGTA				
	*pvl*	*luk*S‐PV1	ATCATTAGGTAAAATGTCTGGACATGATCCA	433	56°C/45	30	(Vittorakis et al. (2023))
		*luk*S‐PV2	GCATCAAGTGTATTGGATAGCAAAAGC				
	SCCmec I	Type I‐F	GCTTTAAAGAGTGTCGTTACAGG	613	54°C/30	30	(Bhowmik et al. (2019); K. Zhang et al. (2005))
		Type I‐R	GTTCTCTCATAGTATGACGTCC				
	SCCmec II	Type II‐ F	CGTTGAAGATGATGAAGCG	398			
		Type II‐ R	CGAAATCAATGGTTAATGGACC				
	SCCmec III	Type III‐ F	CCATATTGTGTACGATGCG	280			
		Type III‐ R	CCTTAGTTGTCGTAACAGATCG				
	SCCmec IVa	Type IVa‐ F	GCCTTATTCGAAGAAACCG	776			
		Type IVa‐R	CTACTCTTCTGAAAAGCGTCG				
	SCCmec IVb	Type IVb‐ F	TCTGGAATTACTTCAGCTGC	493			
		Type IVb‐R	AAACAATATTGCTCTCCCTC				
	SCCmec IVc	Type IVc‐ F	ACAATATTTGTATTATCGGAGAGC	200			
		Type IVc‐R	TTGGTATGAGGTATTGCTGG				
	SCCmec IVd	Type IVd‐ F5	CTCAAAATACGGACCCCAATACA	881			
		Type IVd‐R6	TGCTCCAGTAATTGCTAAAG				
	SCCmec V	Type V‐F	GAACATTGTTACTTAAATGAGCG	325			
		Type V‐R	TGAAAGTTGTACCCTTGACACC				
	SCCmec VI	mecI‐F	CGTTATAAGTGTACGAATGGTTTTTG	126	54°C/60	35	
		mecI‐R	TCATCTGCAGAATGGGAAGTT				
		ccrB4‐F	CGAAGTATAGACACTGGAGCGATA	134			
		ccrB4‐R	GCGACTCTCTTGGCGTTTA				
		IS1272J‐F	GAAGCTTTGGGCGATAAAGA	98			
		IS1272J‐R	GCACTGTCTCGTTTAGACCAATC				

### 2.4 Determination of *Spa* Types

*Spa* typing was performed as described by Aires‐De‐Sousa et al. 2006), with primers and PCR conditions adopted from the original protocol. PCR reactions were prepared using PuRe Taq Ready‐To‐Go PCR Beads (Amersham Biosciences, Freiburg, Germany) in a total volume of 25 μL, containing *nuc*lease‐free water, template DNA, and 10 pmol of each purified primer (spa‐1113F: 5′‐TAA AGA CGA TCC TTC GGT GAG C‐3′; spa‐1514 R: 5′‐CAG CAG TAG TGC CGT TTG CTT‐3′). Thermal cycling conditions consisted of an initial denaturation at 80°C for 5 min, followed by 35 cycles of denaturation at 94°C for 45 s, annealing at 60°C for 45 s, and extension at 72°C for 90 s, with a final extension at 72°C for 10 min. PCR products (20 μL) were subsequently purified using the QIAquick PCR Purification Kit (QIAGEN, Hilden, Germany). Amplicons were then bidirectionally sequenced by a commercial service (Macrogen, Amsterdam, the Netherlands). Resulting sequence data were analyzed in‐house: repeat profiles were assigned using DNASTAR and the Ridom *spa* server (http://www.spaserver.ridom.de/), and the population structure of *S. aureus* was visualized using a Minimum Spanning Tree (MST) based on *spa* repeat‐based clustering. Genetic distances were calculated based on standard alignment‐based clustering algorithms (Harmsen et al. 2003; Mellmann et al. 2007), and the final network was generated using Python (NetworkX library; https://github.com/gundogalp-collab/Staph-MST-Analysis), where node sizes correspond to isolate frequency and branch thicknesses reflect genetic divergence. Detailed computational parameters and a static version of the custom script are also provided in the Supplementary Material.

### 2.5 Statistical Analysis

Statistical analyses were performed using IBM SPSS Statistics software (Version 28.0.1.0; IBM Corp., Armonk, NY, USA). Descriptive statistics were used to describe the distribution and characteristics of the isolates. Pearson's chi‐square test and Fisher's exact test were used to assess the association between categorical variables.

Binary logistic regression analysis was performed to evaluate factors associated with MDR status, PVL gene carriage, and biofilm formation. Statistical significance was defined as a *p*‐value < 0.05. The diversity of spa types among isolates from different sources was assessed using the Hunter–Gaston Discriminatory Index (HGDI), an application of Simpson's Diversity Index (Hunter and Gaston 1988).

The index was calculated using the following formula:

$$D=1-\frac{1}{N\left(N-1\right)}\sum_{j=1}^{s}n_{j}\left(n_{j}-1\right),$$

where **D** denotes the Hunter–Gaston discriminatory index, **1** represents complete discriminatory ability, **N** is the total number of isolates included in the analysis, **s** is the number of distinct spa types, and **n_(j)_** is the number of isolates belonging to the *j*th spa type.

## 3 Results

**Figure 1:**
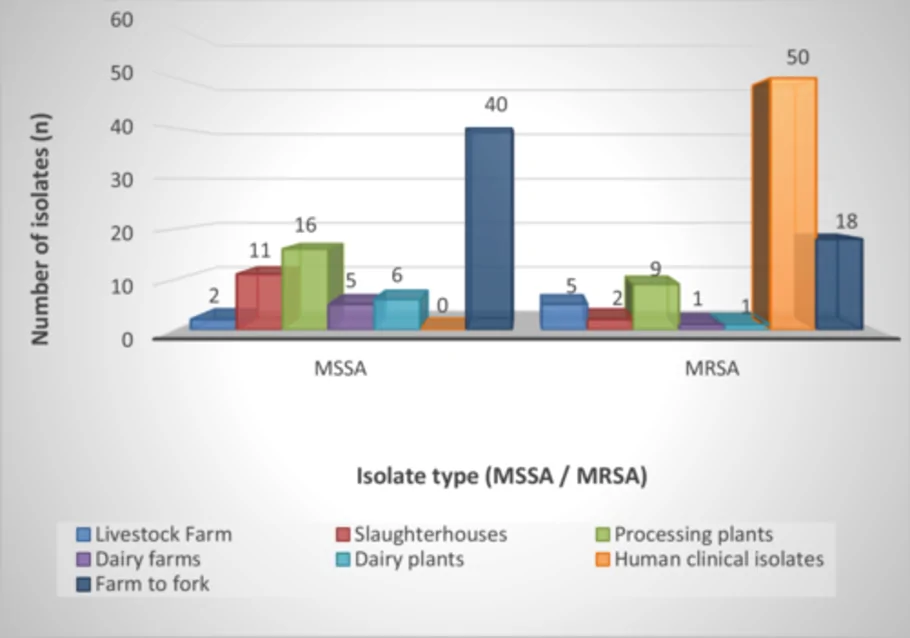
Distribution of MSSA and MRSA isolates obtained from farm‐to‐fork and clinical sources.

A total of 108 *S. aureus* isolates were included in the study, comprising 58 farm‐to‐fork isolates (18 MRSA and 40 MSSA) and 50 clinical human MRSA isolates (Figure 1). Clinical human MRSA isolates originated from wounds (42%), abscesses (22%), blood (20%), and urinary samples (16%) (Table S1).

Farm‐to‐fork isolates were predominantly recovered from the meat chain (45 isolates, 77.6%), whereas only 13 (22.4%) isolates originated from the dairy chain. Of the 18 MRSA isolates, 16 (88.9%) originated from the meat chain and two (11.1%) from the dairy chain. Most meat‐chain isolates originated from processing plants (25/45, 55.5%), followed by slaughterhouses (13/45, 28.8%) and farms (7/45, 15.5%). In the dairy chain, isolates were distributed between processing environments (7/13, 53.8%) and farms (6/13, 46.2%) (Table S1). No significant difference was observed in the distribution of MRSA and MSSA among farm‐to‐fork isolates (*p* > 0.05).

As shown in Table 2, the most prevalent β‐lactam resistance phenotype was resistance to ampicillin and penicillin G, accompanied by β‐lactamase production, detected in 102 isolates (94.4%). Resistance to oxacillin, cefoxitin, and amoxicillin–clavulanic acid was observed in 68 isolates (62.9%). Fluoroquinolone resistance was moderate, with 53 (49.0%) isolates resistant to ciprofloxacin, 51 (47.2%) to moxifloxacin, and 11 (10.1%) to levofloxacin. Fifty‐three (49.1%) isolates were resistant to tetracycline, 47 (43.5%) to erythromycin, 42 (38.9%) to clindamycin, and 33 (30.5%) each to gentamicin and rifampicin. MLSB resistance was observed in 47 isolates (43.5%), comprising 38 (35.1%) cMLSB and 9 (8.3%) MSB phenotypes. However, all isolates were susceptible to amikacin, daptomycin, fosfomycin, fusidic acid, linezolid, nitrofurantoin, teicoplanin, trimethoprim–sulfamethoxazole, and vancomycin.

**Table 2:** Antibiotic resistance profile of *S. aureus* isolates from farm‐to‐fork and human clinical sources.

	Resistance rates (%)			
Antibiotic resistance profile Antibiotic class	Totally (*n* = 108)	Farm‐to‐fork MRSA (*N* = 18)	Farm‐to‐fork MSSA (*N* = 40)	Human clinical MRSA (*N* = 50)
**β‐lactams**				
AMC	68 (62.9%)	18 (100%)	—	50 (100%)
AMP	102 (94.4%)	18 (100%)	34 (85%)	50 (100%)
PEN G	102 (94.4%)	18 (100%)	34 (85%)	50 (100%)
FOX	68 (62.9%)	18 (100%)	—	50 (100%)
OXA	68 (62.9%)	18 (100%)		50 (100%)
**Fluoroquinolones**				
CIP	53 (49%)	2 (11.1%)	4 (10%)	47 (94%)
LEV	11 (10.1%)	2 (11.1%)	3 (7.5%)	6 (12%)
MFX	51 (47.2%)	2 (11.1%)	3 (7.5%)	46 (92%)
**Macrolides**				
E	47 (43.5%)	3 (16.7%)	7 (17.5%)	37 (74%)
**Lincosamides**				
CLI	42 (38.9%)	2 (11.1%)	7 (17.5%)	33 (66%)
**Aminoglycosides**				
GEN	33 (30.5%)	—	—	33 (66%)
**Tetracyclines**				
TE	53(49.1%)	5 (27.8%)	6 (15%)	42 (84%)
**Rifamycins**				
RIF	33 (30.5%)	—	—	33 (66%)
**Phoenix Markers**				
**β‐lactamase positive**	102 (94.4%)	18 (100%)	34 (85%)	50 (100%)
**MLSB phenotype**	47 (43.5%)	3 (16.7%)	7 (17.5%)	37 (74%)
MLSB	38 (35.1%)	2 (11.1%)	7 (17.5%)	29 (58%)
MSB	9 (8.3%)	1 (5.5%)	—	8 (16%)

Abbreviations: AMC, Amoxicillin–Clavulanic Acid; AMP, Ampicillin; CLI, Clindamycin; CIP, Ciprofloxacin; E, Erythromycin; FOX, Cefoxitin; GEN, Gentamicin; LEV, Levofloxacin; MFX, Moxifloxacin; MLSB, Inducible Macrolide–Lincosamide–Streptogramin B Resistance; MSB, Macrolide–Streptogramin B Resistance; OXA, Oxacillin; PEN G, Penicillin G; RIF, Rifampicin; TE, Tetracycline.

A significant association was observed between isolate source and antimicrobial resistance profiles. Clinical MRSA isolates exhibited higher resistance to multiple antimicrobial classes and individual antibiotics than farm‐to‐fork isolates (*p* < 0.001). All MRSA isolates were resistant to β‐lactam antibiotics, whereas MSSA isolates showed resistance to ampicillin and penicillin G at rates of 95.0% each (Table 2). Ciprofloxacin and moxifloxacin resistance remained low among farm‐to‐fork MRSA isolates (11.1% each) and MSSA isolates (10.0% and 7.5%, respectively), but occurred at markedly higher rates among clinical MRSA isolates (94.0% and 92.0%, respectively). Gentamicin and rifampicin resistance were detected only among clinical MRSA isolates (66% each). Resistance to erythromycin, clindamycin, and tetracycline exceeded 65% among clinical MRSA isolates but remained below 29% among farm‐to‐fork MRSA and MSSA isolates. (Table S1, Table 2).

Overall, 59 of 108 (54.6%) isolates exhibited an MDR phenotype, of which 49 (83.1%) were clinical MRSA isolates, 8 (13.6%) were MSSA isolates, and 2 (3.4%) were farm‐to‐fork MRSA isolates (Table 3). MDR was significantly more frequent among MRSA isolates than among MSSA isolates and was associated with broader resistance profiles, including higher numbers of resistant antimicrobial classes and individual antibiotics (*p* < 0.001). Clinical MRSA isolates exhibited more extensive MDR profiles, most frequently involving resistance to seven antimicrobial classes and 12 individual antibiotics (13/50, 26%), followed by resistance to five classes and 10 antibiotics (8/50, 16%). In contrast, MDR‐positive farm‐to‐fork isolates generally displayed resistance to three to five antimicrobial classes and four to eight antibiotics (Table 3).

**Table 3:** MDR frequencies of *S. aureus* isolates from farm‐to‐fork and human clinical sources.

No. of antibiotic class/No. of antibiotics	Isolate code	*S. aureus*	MDR Profile	Frequencies of MDR isolates (%)		
				Farm‐to‐fork		
				MRSA (*n* = 18)	MSSA (n = 40)	Human clinical MRSA (*n* = 50)
3/4	3, 12, 33, 43	MSSA	AMP, CLI, E, PEN G		4 (10%)	
3/6	38	MSSA	AMP, CIP, LEV, MFX, PEN G, TE		1 (2.5%)	
3/7	37	MRSA	AMC, AMP, CLI, E, FOX, OXA, PEN G	1 (5.5%)		
4/1	63	MRSA	AMC, AMP, CIP, FOX, GEN, LEV, MFX, OXA, PEN G, RIF			1 (2%)
4/5	19	MSSA	AMP, CIP, CLI, E, PEN G		1 (2.5%)	
4/5	34, 47	MSSA	AMP, CLI, E, PEN G, TE		2 (5%)	
4/8	27, 77	MRSA	AMC, AMP, CLI, E, FOX, OXA, PEN G, TE	1 (5.5%)		1 (2%)
4/8	93	MRSA	AMC, AMP, CIP, FOX, OXA, PEN G, RIF, TE			1 (2%)
4/9	67	MRSA	AMC, AMP, CIP, FOX, GEN, MFX, OXA, PEN G, RIF			1 (2%)
4/9	83, 108	MRSA	AMC, AMP, CIP, CLI, E, FOX, MFX, OXA, PEN G			2 (4%)
4/9	102	MRSA	AMC, AMP, CIP, FOX, GEN, MFX, OXA, PEN G, TE			1 (2%)
4/9	103	MRSA	AMC, AMP, E, FOX, GEN, MFX, OXA, PEN G, RIF			1 (2%)
4/9	106	MRSA	AMC, AMP, CIP, CLI, FOX, MFX, OXA, PEN G, TE			1 (2%)
5/1	59, 64, 66, 71, 81, 84, 97, 104	MRSA	AMC, AMP, CIP, CLI, E, FOX, MFX, OXA, PEN G, TE			8 (16%)
5/1	60,105	MRSA	AMC, AMP, CIP, E, FOX, MFX, OXA, PEN G, RIF, TE			2 (4%)
5/1	69	MRSA	AMC, AMP, CIP, CLI, FOX, GEN, MFX, OXA, PEN G, TE			1 (2%)
5/1	70	MRSA	AMC, AMP, CIP, E, FOX, MFX, OXA, PEN G, GEN, RIF			1 (2%)
5/1	73, 94	MRSA	AMC, AMP, CIP, FOX, GEN, MFX, OXA, PEN G, RIF, TE			2 (4%)
5/11	82	MRSA	AMC, AMP, CIP, FOX, GEN, LEV, MFX, OXA, PEN G, RIF, TE			1 (2%)
5/11	88	MRSA	AMC, AMP, CIP, CLI, FOX, GEN, LEV, MFX, OXA, PEN G, RIF			1 (2%)
5/9	107	MRSA	AMC, AMP, FOX, GEN, LEV, OXA, PEN G, RIF, TE			1 (2%)
6/10	79	MRSA	AMC, AMP, CIP, E, FOX, GEN, OXA, PEN G, RIF, TE			1 (2%)
6/11	65	MRSA	AMC, AMP, CIP, CLI, E, FOX, MFX, OXA, PEN G, RIF, TE			1 (2%)
6/11	74, 87	MRSA	AMC, AMP, CIP, CLI, E, FOX, GEN, MFX, OXA, PEN G, TE			2 (4%)
6/11	75	MRSA	AMC, AMP, CIP, CLI, FOX, GEN, MFX, OXA, PEN G, RIF, TE			1 (2%)
6/11	86, 89, 99	MRSA	AMC, AMP, CIP, E, FOX, GEN, MFX, OXA, PEN G, RIF, TE			3 (6%)
7/12	61, 62, 68, 80, 85, 90, 91, 92, 95, 96, 98, 100, 101	MRSA	AMC, AMP, CIP, CLI, E, FOX, GEN, MFX, OXA, PEN G, RIF, TE			13 (26%)
7/13	72, 78	MRSA	AMC, AMP, CIP, CLI, E, FOX, GEN, LEV, MFX, OXA, PEN G, RIF, TE			2 (4%)

Abbreviations: AMC, Amoxicillin–Clavulanic Acid; AMP, Ampicillin; CLI, Clindamycin; CIP, Ciprofloxacin; E, Erythromycin; FOX, Cefoxitin; GEN, Gentamicin; LEV, Levofloxacin; MFX, Moxifloxacin; OXA, Oxacillin; PEN G, Penicillin G; RIF, Rifampicin; TE, Tetracycline.

**Figure 2:**
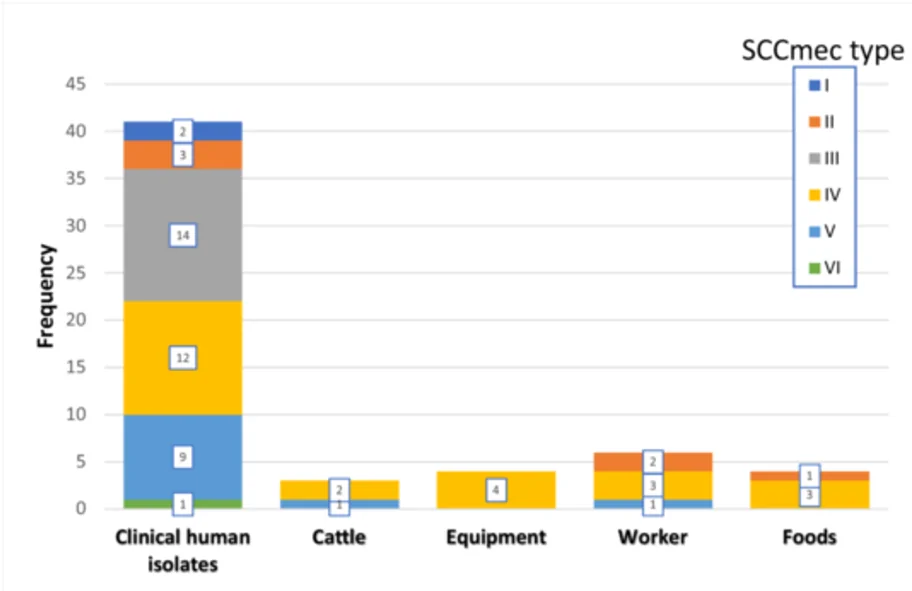
Distribution of SCCmec types among MRSA isolates from clinical human, animal, food of animal origin, and food‐related environments.

SCCmec types (I–VI) were identified in 58 (85.2%) of the 68 MRSA isolates, with type IV being predominant (24/58, 41.4%), followed by III (14/58, 24.2%), V (11/58, 19.0%), II (6/58, 10.3%), I (2/58, 3.4%), and VI (1/58, 1.7%). Among farm‐to‐fork MRSA isolates, SCCmec type IVa was predominant (12/18, 66.7%), followed by type II (3/18, 16.7%) and type V (2/18, 11.1%). In 50 clinical MRSA isolates, SCCmec typing identified six types (I–VI) in 41 isolates, while nine isolates were untypeable. Among the typed isolates, type III was most frequent (14/41, 34.1%), followed by type IV (12/41, 29.3%), type V (9/41, 22.0%), type II (3/41, 7.3%), and types I and VI (2/41, 4.9% and 1/41, 2.4%, respectively) (Figure 2, Table S1). The binary logistic regression model showed that SCCmec IV variants were associated with 5.6‐fold higher odds of MDR positivity compared with MSSA (OR = 5.57, 95% CI: 1.77–17.52, *p* = 0.003). In addition, the number of antibiotics to which isolates were resistant varied significantly among SCCmec groups (*p* < 0.001), with non‐IV and untypeable isolates showing the highest levels of antimicrobial resistance.

**Figure 3:**
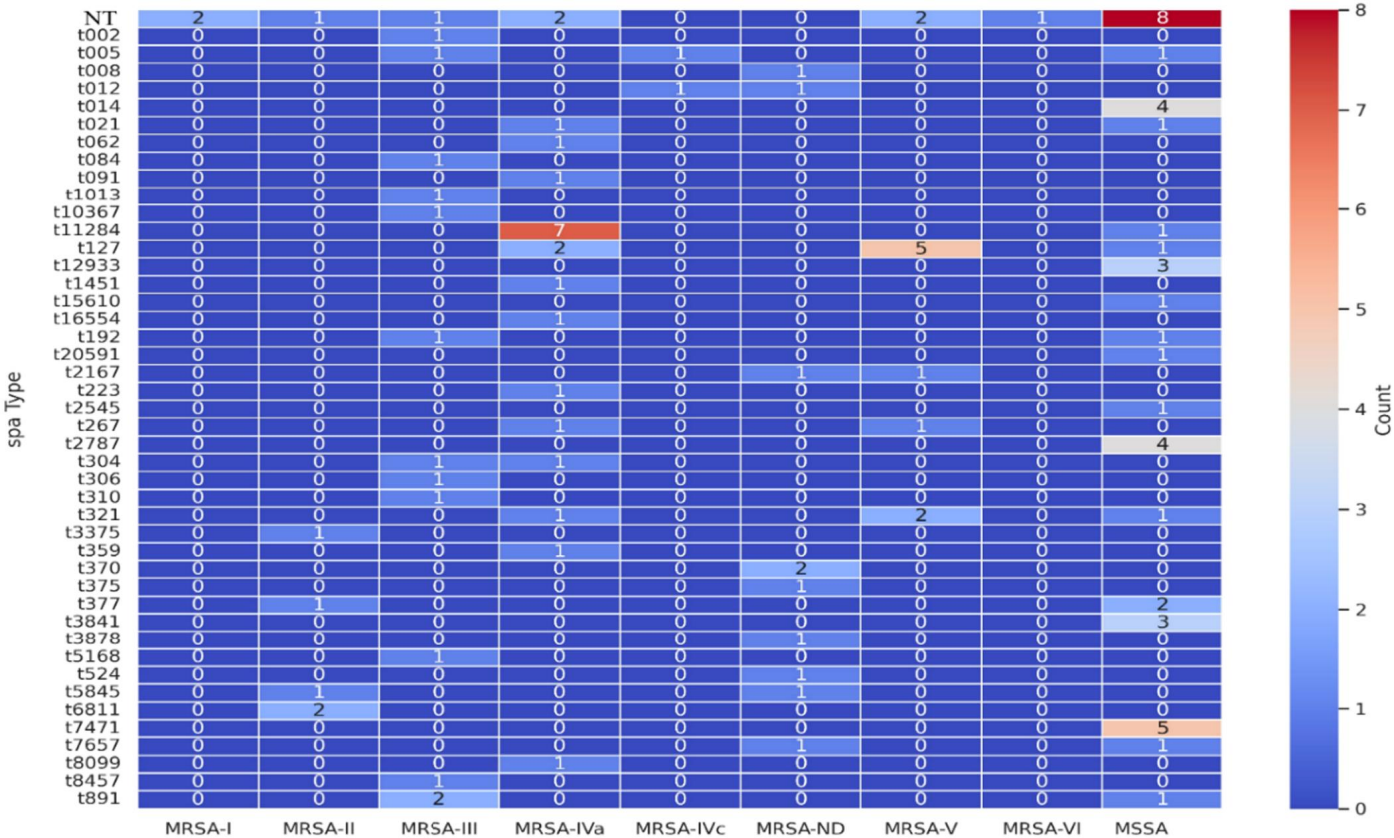
The heatmap illustrates the frequency distribution of identified *S. aureus spa* types across MRSA clonal categories and MSSA isolates. Each row represents a *spa* type, and each column represents a clonal group (MRSA‐I, MRSA‐II, MRSA‐III, MRSA‐IVa, MRSA‐IVc, MRSA‐V, MRSA‐VI, MRSA‐ND) and MSSA. The numbers within cells indicate the number of isolates (n). The color gradient ranges from white (no isolate detected) to dark red (highest frequency), with increasing color intensity corresponding to higher counts. A total of *n* = 108 isolates were included in the analysis, comprising MRSA (*n *= 68) and MSSA (*n* = 40) isolates. MRSA clonal classification was based on *SCCmec* typing results. MRSA‐ND indicates MRSA isolates for which *SCCmec* type could not be determined. MSSA represents methicillin‐susceptible *S. aureus*. The heatmap provides a descriptive visualization of spa type diversity and their distribution among clonal lineages; no statistically significant association was observed between spa type distribution and MRSA clonal groups (*p* > 0.05).

**Figure 4:**
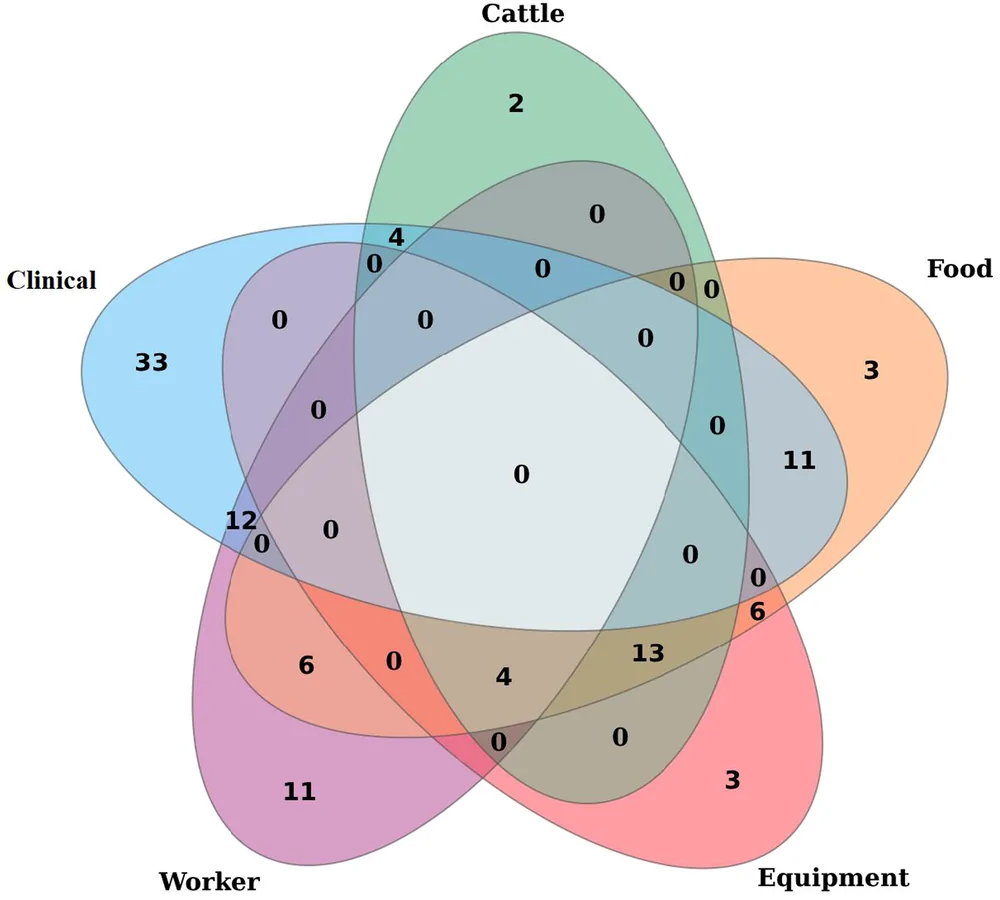
Venn diagram showing the number of shared and unique *spa* types among MRSA isolates from animals, workers, food of animal origin, equipment, and clinical settings. The overlapping numbers represent all isolates sharing the same *spa* types across multiple sources, while the non‐overlapping numbers indicate isolates unique to each source. The numbers in each section correspond to the count of unique or shared *spa* types, and the total sum of isolates may differ from the individual counts due to overlaps between categories. Cattle represent spa types specific to animal‐derived isolates, Worker refers to spa types found in workers handling animals or food products, Food indicates *spa* types found in food of animal origin, Equipment represents spa types from equipment used in animal and food processing environments, and clinical indicates *spa* types from clinical settings. No statistically significant difference was observed in the distribution of *spa* types across the sources (*p* > 0.05).

Of the 108 *S. aureus* isolates, 91 (84.3%) were spa‐typable, yielding 44 distinct repeat‐pattern types (Figure 3). The most common spa types were t127 and t11284, each detected in 8 isolates (8.8%), followed by t7471 with 5 isolates (5.5%). Both farm‐to‐fork and hospital isolates showed high spa type diversity, with HGDI values of 0.936 and 0.958, respectively. Nearly half of the isolates (52/108, 48.1%) carried spa types confined to a single source, whereas the remaining isolates (56/108, 51.9%) were shared across multiple sources, most frequently involving clinical–worker, clinical–food and cattle–equipment–food combinations (Figure 4). The distribution of *spa* groups differed significantly according to isolate source category (*p* = 0.005). *spa* type t11284 was detected exclusively among farm‐to‐fork isolates, whereas t127 was predominantly identified among clinical human isolates. Several common *spa* types, including t11284, t127, t321 and t014, occurred in both MRSA and MSSA and across different host categories. *Spa* types t005, t891, t267, t304, and t192 were shared between clinical isolates and workers, whereas t014, t7471, and t11284 were found across cattle, equipment, and food. Seventeen isolates (15.7%) possessed novel repeat patterns, as detailed in Table 4.

**Table 4:** The distribution of MRSA isolates and their associated SCC*mec* types, sharing the same *spa* types, from different sources.

*Spa* type	*Spa* repeat	SCC*mec* type	Source (n)	Origin of samples
t11284	07‐23‐12‐34‐12‐12‐12‐12‐23‐02‐12‐23	IVa	Cattle Nasal Swab	Livestock Farm
			Manger (2)	Livestock Farm
			Carcass	Slaughterhouse
			Filling Machine (2)	Meat Plant
			Sausage	Meat Plant
t321	07‐23‐16‐34‐33‐13	IVa	Cattle Nasal Swab	Slaughterhouse
		V	Blood (2)	Clinic
NT*	16‐17‐39‐16‐23‐17‐17‐17	II	Urinary Tract	Clinic
		IVa	Wound	
t6811	07‐23‐21‐16‐34‐33	II	Worker	Meat Plant
			Pastirma	
t304	11‐10‐21‐17‐34‐24‐34‐22‐25	IVa	Worker	Meat Plant
		III	Wound	Clinic
t5845	16‐34	II	Worker	Meat Plant
		ND	Wound	Clinic
t267	07‐23‐12‐21‐ 17‐ 34‐34‐34‐33‐34	V	Worker	Meat Plant
		IVa	Urinary Tract	Clinic
t005	26‐23‐13‐23‐31‐05‐17‐25‐17 ‐25‐16‐28	IVc	Abscess	Clinic
		III	Wound	
t127	07‐23‐21‐16‐34‐33‐13	V	Wound (2)	Clinic
			Abcess (3)	
		IVa	Blood	
			Wound	
t012	15‐12‐16‐02‐16‐02‐25‐17‐24‐24	ND	Urinary Tract	Clinic
		IVc	Wound	
t370	09‐34‐17‐34‐16‐34	ND	Wound	Clinic
			Blood	
t2167	17‐34‐17‐20‐17‐12‐17‐16	ND	Wound	Clinic
		V	Wound	
t891	26‐23‐13‐23‐31‐05‐17‐25‐17‐25‐28	III	Wound (2)	Clinic

*Note:* n: number of isolates sharing the same *spa* type obtained from the indicated source, * NT: Nontypeable; although it has repeats, it does not resemble any other *spa* type, ND: Not detected.

**Figure 5:**
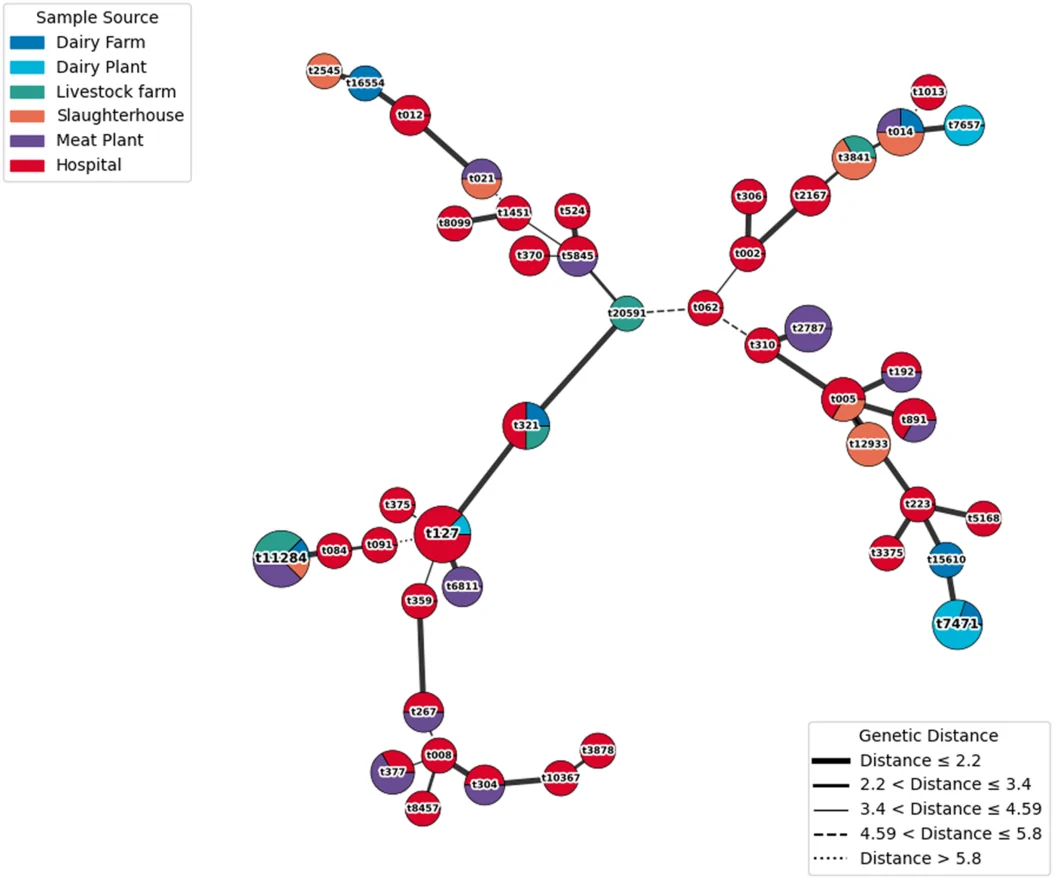
The network was constructed using a *spa* repeat‐based clustering analysis encompassing the complete dataset. Each node represents a distinct *spa* genotype, with the node area scaled proportionally to the total number of isolates (square root of frequency). Pie charts within the nodes depict the relative distribution of isolates across diverse sample sources, explicitly highlighting the multi‐sectoral presence of central hubs such as t127 and t005. The thickness and style of the connecting branches indicate the genetic divergence, calculated as the Levenshtein edit distance between repeat sequences. Thicker solid lines represent tight genetic proximity (distance ≤ 2.2), whereas dashed and dotted lines indicate progressively greater genetic distances, thereby aiding in the visual interpretation of potential epidemiological links.

MST analysis based on *spa* repeat profiles revealed a heterogeneous distribution of *spa* types with varying repeat‐based relatedness among the identified isolates (Figure 5). Several *spa* types comprised multiple isolates originating from diverse sources and exhibited varying repeat‐based distances within the network. *Spa* types t11284, t127, and t321 were among the nodes with higher isolate frequency and source diversity. Hospital‐derived isolates were distributed across multiple *spa* types throughout the network rather than being confined to a single cluster. Clinical‐associated *spa* types were frequently positioned in close proximity to food‐ and farm‐related isolates within the MST network, including t304, t377, t267, t11284, t6811, t321, t005, t891, and t192. Notably, the central hub t127 contained isolates from multiple sources and was connected to several adjacent farm‐related meat plant *spa* types at short repeat‐based distances (≤ 2.2). Similarly, *spa* type t005 included isolates from both hospital and slaughterhouse sources and was positioned within a mixed‐source cluster characterized by short repeat‐based distances (≤ 2.2). In contrast, MDR frequency did not differ significantly among spa types (χ^2^ = 3.070, df = 3, *p* = 0.381), with the highest proportion observed in t127 isolates (87.5%).

Forty‐one isolates (38%) were positive for the pvl gene, including 32 MRSA and 9 MSSA isolates, with 20 derived from farm‐to‐fork sources and 21 from clinical isolates. PVL gene carriage was significantly higher in MRSA than in MSSA isolates (*p* = 0.007) and showed a strong association with SCCmec type (*p* < 0.001), being detected exclusively in isolates carrying SCCmec IVa, IVc, or V variants. The highest prevalence was observed in SCCmec IVa isolates (91.7%), whereas no PVL positivity was detected in SCCmec types I, II, or III. Logistic regression analysis confirmed a strong association between SCCmec IV and PVL carriage (OR = 39.11, 95% CI: 7.70–198.72, *p* < 0.001). In addition, PVL positivity differed significantly among spa types (*p* = 0.020), with the highest rates observed in t11284 and t127 (75.0% and 62.5%, respectively).

The *tst*‐1 gene was detected in 10 isolates (9.3%), including four farm‐to‐fork and six human‐derived isolates. Enterotoxin genes were identified in nine isolates (8.3%). In clinical MRSA, *sea* was the most frequent (*n* = 4) and consistently co‐occurred with *pvl* gene, mainly in wound isolates and once in a blood sample. The *sed* gene was detected in both dairy‐associated MSSA and a single clinical MRSA abscess isolate, whereas *seb* occurred only in clinical MRSA (Table 5).

**Table 5:** Distribution of virulence genes and biofilm‐related genotypic and phenotypic characteristics among MRSA (*n* = 68) and MSSA (*n* = 40) isolates (*n* = 108 total).

Virulence category	Gene	Total isolates (*n* = 108)	MRSA (*n* = 68)	MSSA (*n* = 40)	Associated *spa* types	Associated SCCmec types
**Toxic shock–related**	*tst‐1*	10 (9.3%)	7 (10.2%)	3 (7.5%)	t6811, t10367, t192, t3375, t084, t008, t3841, t12933, t321	I, II, III
**Enterotoxins**	*sea*	4 (3.7%)	4 (5.8%)	—	t127, t223, t375	IVa
	*seb*	2 (1.9%)	2 (2.9%)		t2167, t891	V, III
	*sed*	3 (2.8%)	1 (1.4%)	2 (5%)	t7471 (MSSA), t5168(MRSA)	III
**Biofilm‐associated**	*icaA*	—	—	—	—	—
	*icaD*	20 (18.5%)	17 (25%)	3 (7.5%)	t321, t014, t127, t192, t062, t5845, t891, t8457, t310, t005, t370, t304, t091	I‐III, IVa, V
	*icaA+icaD*	33 (30.5%)	13 (19.1%)	20 (50%)	t014, t7471, t16554, t11284, t7657, t127, t3841, t20591, t12933, t021, t5845, t192, t2787, t012, t3878, t2167	I‐III, IVa, V
**PVL**	*luk*S*‐PV/luk*F*‐PV*	41 (38%)	32 (47%)	9 (22.5%)	t127, t370, t012, t5845, t223, t091, t005, t359, t375, t321, t1451, t8099, t524, t062, t267, t11284, t321, t021, t304, t267, t7657, t014, t15610, t3841, t20951, t021, t2787, t12933	IVa, IVc, V
**Biofilm phenotype**	—	49 (45,3%)	23 (33.8%)	26 (65%)	t012, t014, t021, t11284, t127, t12933, t16554, t192, t20591, t2167, t267, t2787, t304, t321, t370, t3841, t3878, t5845, t7471, t7657, t891	I‐III, IVa, V

Biofilm production was detected in 49 isolates (45.3%), including 19 strong (17.6%), 18 moderate (16.7%), and 12 weak (11.1%) producers. All strong and most moderate biofilm‐forming isolates carried both *icaA* and *icaD*, whereas weak producers typically carried *icaD* alone (Table S1, Table 5). Strong biofilm formation was significantly more frequent among farm‐to‐fork isolates than among clinical human isolates (*p* < 0.001). Univariate analysis showed a significant association between biofilm formation and MDR status (*p* < 0.001), with strong biofilm production more frequently observed in non‐MDR isolates compared with MDR‐positive isolates (56.1% vs. 20.9%). However, this association was not confirmed in logistic regression analysis (OR = 0.45, 95% CI: 0.16–1.26, *p* = 0.128).

## 4 Discussion

*S. aureus* is a versatile pathogen capable of colonizing both humans and animals, with clonal lineages showing varying levels of host adaptation (Tang et al. 2024). However, host specificity does not preclude interspecies transmission and bidirectional exchange between humans and livestock (Pan et al. 2023). SCCmec typing is crucial for MRSA source tracking and reflects the distribution of resistance‐associated genetic elements (Hesari et al. 2020), while *spa* typing offers complementary information on clonal backgrounds and population structure (Banaszkiewicz et al. 2023).

In the present study, SCCmec III predominated among clinical isolates, whereas SCCmec IVa was most frequent in farm‐to‐fork isolates, consistent with previous reports (Hofstetter et al. 2024; Özdemir 2022; Szumlanski et al. 2022; Tavsanli and Cibik 2022). SCCmec types IV/V were also detected in clinical isolates, while II/V were found in farm‐to‐fork isolates. Although SCCmec IV/V are commonly associated with CA‐MRSA and SCCmec I–III with HA‐MRSA in the literature (S. Wu et al. 2019), our findings suggest the coexistence of different SCCmec lineages across both clinical and farm‐to‐fork isolates.

We observed high spa diversity, with t127 and t11284 being the most prevalent types (each 8.8%). Spa type t127 has been frequently associated with the ST1/CC1 lineage in previous studies and reported from human, food, and animal sources (Arfaoui et al. 2022; Gehrke et al. 2023; Jones et al. 2021; X. Li et al. 2022; Papadopoulos et al. 2019; Silva et al. 2020). *Spa* type t127 predominated among clinical MRSA isolates, mainly associated with SCCmec V and less frequently IVa, contrasting with earlier reports from Türkiye of *spa*‐t030/SCCmec III (Alp et al. 2009; Kırca Yılmaz et al. 2014), suggesting temporal or regional variation in MRSA population structure.

MRSA IVa–t11284 was detected across different stages of the farm‐to‐fork continuum, suggesting its presence in multiple production‐associated settings. In contrast, Özdemir (2022) reported spa type t091 as predominant among MSSA from chicken meat and ground beef, highlighting differences in spa type distribution between MRSA and MSSA populations within food‐related sources. Notably, t11284 has been rarely reported and was previously identified only once in a clinical isolate from Germany within CC84 (Becker et al. 2017), indicating that its detection in the present study may extend its known ecological context. In addition, MRSA isolates from a meat plant worker and a pastirma sample shared identical SCCmec II and spa type t6811 profiles, indicating the presence of the same genotype in both human and food sources within the processing environment. This spa type has also been reported in food industry workers, mastitis milk, and clinical isolates associated with the ST1–SCCmec V/CC1 lineage (Bonsaglia et al. 2018; Boswihi et al. 2020; Çakici 2019; Goudarzi et al. 2020).

As illustrated by the spa repeat–based MST, spa types t304 and t267, which have been widely reported in animal, food, and human isolates across earlier studies (Alfouzan et al. 2019; Bencardino et al. 2017; Boswihi et al. 2024; Khalil et al. 2012; Negrete‐González et al. 2020; Papadopoulos et al. 2019; Pauly et al. 2019), were clustered together with both meat plant worker and clinical isolates. Similarly, spa types t192, t891, and t005 were shared between worker‐derived MSSA and clinical MRSA isolates and formed closely related clusters within the MST network. These spa types have previously been associated with CC22‐related lineages circulating in both healthcare and community settings (Goudarzi et al. 2018; Oosthuysen et al. 2014; Uzunović et al. 2013). Overall, the spa‐based MST revealed interspersed clustering of isolates from clinical and worker‐associated sources, suggesting shared spa types between these groups. *Spa* types t005, t012, t084, t127, t267, t359, t377, and t5168 have also previously been reported in Türkiye (Dündar et al. 2016; Özdemir 2022; Tavsanli and Cibik 2022; Kırca Yılmaz et al. 2014).

Clinical MRSA isolates exhibited multidrug resistance, with resistance to most tested antibiotic classes, whereas farm‐to‐fork isolates were predominantly resistant to β‐lactams. Notably, gentamicin and rifampicin resistance were detected exclusively among clinical isolates, similar to findings previously reported for hospital‐associated MRSA populations (Hu et al. 2021; Liang et al. 2019; Rønning et al. 2025). These findings indicate broader co‐resistance patterns among clinical MRSA isolates, which may reflect antimicrobial selective pressure in healthcare settings (Luo et al. 2018; Tang et al. 2025).

The SCCmec III clinical MRSA isolates exhibited concurrent resistance to multiple non‐β‐lactam antibiotic classes, particularly fluoroquinolones, aminoglycosides, tetracyclines, and rifampicin, similar to those reported by Alseqely et al. (2021) and Rønning et al. (2025). Macrolide‐lincosamide resistance was also higher among SCCmec III clinical isolates than among farm‐to‐fork isolates, with 50.0% and 28.6% exhibiting the MLSB and MSB phenotypes, respectively. Similarly, ciprofloxacin (94%) and moxifloxacin (92%) resistance rates among clinical MRSA isolates markedly exceeded the resistance observed for levofloxacin (12%), whereas fluoroquinolone resistance among farm‐to‐fork isolates remained limited (≤ 11.1%). This resistance profiles observed among clinical isolates are compatible with those previously reported for hospital‐associated MRSA lineages (Alseqely et al. 2021; Hu et al. 2021; Liang et al. 2019; X. Wu et al. 2023) and may reflect differences in ecological niches, genetic backgrounds, and antimicrobial selective pressures between clinical and farm‐to‐fork environments. Despite the extensive spa diversity and genetic overlap observed among sources, antimicrobial resistance patterns remained largely source‐associated rather than spa‐type‐associated. Additionally, previous studies have suggested that antimicrobial pressure and environmental adaptation may contribute to the diversification of *S. aureus* lineages (Mollerup et al. 2022; Pan et al. 2023; Schouls et al. 2009; Turner et al. 2019; Uhlemann et al. 2017). However, despite the extensive spa diversity and genetic overlap observed among sources in the present study, antimicrobial resistance patterns appeared to be more strongly associated with isolate source than with spa type.

MRSA isolates carrying the pvl gene predominantly belonged to SCCmec IV and V backgrounds and exhibited considerable spa diversity across both clinical and farm‐to‐fork sources, consistent with the epidemiological characteristics of community‐associated MRSA lineages (Shohayeb et al. 2023; Szumlanski et al. 2022). Given the established association of the *pvl* gene with severe skin and soft tissue infections, the occurrence of *pvl* gene‐positive *S. aureus* isolates in both clinical and farm‐to‐fork sources may pose a public health concern, as it suggests that clinically relevant virulence determinants are present across interconnected compartments of the human–animal–food continuum. Furthermore, the presence of the *pvl* gene in both MRSA and MSSA isolates indicates that this virulence‐associated determinant is not restricted to methicillin‐resistant lineages, aligning with previous reports describing its distribution among diverse genetic backgrounds and ecological settings (Cuny et al. 2015; Funaki et al. 2019; Nor Amdan et al. 2024; Sadat et al. 2022). Notably, most pvl gene‐positive clinical MRSA isolates in the present study also exhibited MDR, indicating the coexistence of clinically relevant virulence and resistance determinants within the same genetic backgrounds. Similarly, pvl‐positive MRSA lineages have been reported to frequently carry antimicrobial resistance determinants alongside their intrinsic β‐lactam resistance, thereby complicating infection prevention and control efforts (Macedo‐Viñas et al. 2014; Sadat et al. 2022; Szumlanski et al. 2022).

TSST‐1 is a pyrogenic superantigen associated with severe systemic manifestations, including fever, hypotension, and multi‐organ involvement (Zheng et al. 2020). The *tst*‐1 gene was detected at a low frequency (9.3%) and was present particularly in worker‐derived isolates, with additional detection in both clinical and farm‐to‐fork sources. This distribution suggests that *tst*‐positive *S. aureus* lineages may occur across the human–animal–food interface, while variations in *tst* prevalence may reflect differences in environmental conditions and regulatory mechanisms affecting toxin expression (Zhao et al. 2019). Similar low prevalences have been reported in food handlers, food‐contact surfaces, and dairy products (Arcuri et al. 2010; Aung et al. 2017; Ghasemi and Mahdavi 2018; Sospedra et al. 2012).

In the present study, enterotoxin genes were detected in 8.3% of isolates, with *sea* being the most frequent among clinical MRSA isolates. Notably, all *sea*‐positive clinical isolates also carried the pvl gene, indicating co‐occurrence of virulence genes within the same isolates, which may contribute to their pathogenic potential. Similar co‐carriage patterns have previously been reported among community‐associated *S. aureus* lineages and have been associated with synergistic effects that may enhance virulence and host tissue damage (Sadat et al. 2022; Vittorakis et al. 2023). On the other hand, *sed* gene was detected at low frequency in both dairy‐associated MSSA and a clinical MRSA isolate, with relevance to food safety due to the heat stability of staphylococcal enterotoxins (Li et al. 2019; Neelam et al. 2022).

Biofilm formation was detected in 45.3% of isolates, with strong producers predominantly observed among farm‐to‐fork MSSA isolates, whereas clinical MRSA isolates generally exhibited weak or moderate biofilm phenotypes despite higher multidrug resistance. This suggests that biofilm formation and antimicrobial resistance are not always co‐selected and may reflect distinct adaptive responses across different ecological sources. It has been reported that biofilm capacity is driven by clonal background, environmental factors, and regulatory pathways rather than methicillin resistance status alone (Ghasemian et al. 2016; Grinholc et al. 2007; Hernández‐Cuellar et al. 2023; Khasawneh et al. 2020).

Biofilm phenotypes showed variability across spa types. In particular, t11284 exhibited heterogeneous profiles ranging from strong to negative biofilm formation, while t7471 was mainly associated with strong and moderate biofilm production. Although t127 lineages have been reported to carry multiple biofilm‐associated genes (Cai et al. 2026), the isolates in the present study displayed heterogeneous biofilm‐forming phenotypes ranging from negative to moderate. Notably, the presence of the *icaD* gene among isolates with weak or no biofilm production is consistent with previous studies suggesting that the presence of biofilm‐associated genes alone is insufficient to predict phenotypic expression, and that biofilm formation is heavily influenced by additional regulatory and strain‐specific factors (Hernández‐Cuellar et al. 2023; McCarthy et al. 2015).

Most strong biofilm‐forming isolates carried both *icaA*/*icaD*, and several also harbored additional virulence genes, such as pvl and/or *tst*‐1. Previous studies have similarly reported co‐occurrence of biofilm‐ and virulence‐associated determinants among *S. aureus* isolates from food and animal sources (Chowdhury et al. 2025; Tang et al. 2025). These findings suggest that some farm‐to‐fork isolates may combine traits associated with persistence and potential pathogenicity. Biofilm‐forming *S. aureus* has been reported to exhibit increased tolerance to disinfectants, potentially contributing to contamination and cross‐contamination in food production environments (Gutiérrez et al. 2012; Silva‐De‐jesus et al. 2022; Song et al. 2024). Of note, linezolid remained fully effective against all isolates, despite the presence of virulence determinants, highlighting its continued clinical relevance.

This study provides comprehensive data on the molecular, antimicrobial, and genotypic features of *S. aureus* from farm‐to‐fork and human clinical sources within a One Health framework. However, several limitations should be acknowledged. Firstly, although farm‐to‐fork sampling was performed through traceable production chains, this cross‐sectional study provides only a snapshot of *S. aureus* populations across farm‐to‐fork and clinical sources at a single point in time. This design does not capture temporal or seasonal dynamics, which limits the ability to infer the directionality of transmission or establish definitive causal links between different sources. While shared *spa* types were identified across human and farm‐to‐fork isolates, the lack of longitudinal data prevents the determination of whether these overlaps reflect recent interspecies transmission events or long‐standing stable populations in specific niches. Secondly, sampling was restricted to two selected high‐capacity dairy and meat facilities that voluntarily granted permission, which limited the representation of the full diversity of *S. aureus* circulating in other regions and production systems. Furthermore, phenotypically identical isolates may still exhibit underlying heterogeneity, and therefore, the analysis of a single isolate per sample may have limited the detection of minor strain subpopulations within the same sample.

Third, although *spa* and SCCmec typing are well‐established epidemiological tools, the lack of higher‐resolution genomic techniques such as MLST or WGS limited the ability to resolve fine‐scale clonal relationships and trace definitive transmission pathways. Apart from that, alternative methicillin resistance genes, including *mecC* and *mecB*, were not investigated, which may have limited comprehensive genotypic characterization of MRSA isolates. Despite these limitations, this study provides comprehensive insights into the potential epidemiological connections and genetic overlap across the human–animal–environment interface, encompassing both dairy and meat production chains alongside human clinical settings. These findings provide valuable baseline data to guide future longitudinal and genomic investigations.

While *spa* typing alone does not confirm definitive clonal relatedness (Mollerup et al. 2022), the detection of shared spa types across different sources in this study corresponds to patterns previously described in overlapping *S. aureus* populations within interconnected One Health‐associated environments (Pan et al. 2023; Turner et al. 2019; Uhlemann et al. 2017). Previous reports have also demonstrated substantial concordance between spa typing, PFGE, and genome‐based approaches (Alkharsah et al. 2019; Hallin et al. 2007; Koreen et al. 2004).

## 5 Conclusions

This study provides an integrated molecular and phenotypic characterization of *S. aureus* isolates recovered from farm‐to‐fork and human clinical sources in Türkiye. The coexistence of diverse spa and SCCmec types across different sources indicates substantial genetic heterogeneity and suggests that some genetic lineages may be shared among human, animal, and food‐related populations. Antimicrobial resistance profiles differed by source, with broader multidrug resistance observed among clinical MRSA isolates, while farm‐to‐fork isolates were mainly resistant to β‐lactams. However, resistance patterns were not consistently associated with specific spa types, which suggests that antimicrobial resistance may be influenced more by ecological and selective pressures than by clonal background alone. Virulence‐associated determinants, including *pvl*, *tst*‐1, and enterotoxin genes, were detected at variable frequencies among isolates. PVL was the most frequently detected virulence gene and was present in both clinical and farm‐to‐fork isolates, whereas enterotoxin genes were detected only in a limited number of isolates. The detection of toxin‐associated genes outside hospital settings, together with the presence of biofilm‐forming strains in processing environments, may be relevant for public health and food safety. These findings support the value of coordinated surveillance across human, animal, and food sectors within a One Health framework. Future studies using whole‐genome sequencing and longitudinal sampling are required to better resolve clonal relationships and clarify epidemiological linkages at the human–animal–environment interface.

## Author Contributions

**Pinar Sagiroglu:** investigation, methodology, writing – review and editing. **Dursun Alp Gundog:** investigation, visualization, formal analysis, resources. **Candan Gungor:** investigation, visualization, formal analysis, resources. **Kursat Koskeroglu:** investigation, resources. **Mustafa Altay Atalay:** formal analysis, writing – review and editing, investigation, resources. **Adalet Dishan:** investigation. **Yeliz Ucar:** Investigation. **Aytac Akcay:** data curation, formal analysis, visualization. **Huseyin Burak Disli:** investigation. **Harun Hizlisoy:** investigation. **Mukaddes Barel:** Investigation. **Tekin Kececi:** investigation. **Nurhan Ertas Onmaz:** conceptualization, methodology, funding acquisition, project administration, writing – original draft, writing – review and editing.

## Ethics Statement

Our surveillance study was approved by both the Human Research Ethics Committee (Reference: 2019/155) and the Local Ethics Committee for Animal Experiments (Reference:19/036) of Erciyes University, Kayseri Türkiye.

## Consent

Informed consent was obtained from all individual participants included in the study.

## Conflicts of Interest

The authors declare no conflicts of interest.

## Supporting information


Supporting File 1

Supporting File 2

Supporting File 3

Supporting File 4

## Data Availability

The data that support the findings of this study are available on request from the corresponding author. The data are not publicly available due to privacy or ethical restrictions.
