# Supplementary material for: Genetic Diversity and Epidemiological Overlap of Staphylococcus aureus at the Animal–Food–Environment–Human Interface Within a One Health Framework
Source: Microbiologyopen. 2026 Sep 3;15(5):e70397. doi: 10.1002/mbo3.70397 (PMC13542186; doi:10.1002/mbo3.70397)
Supplement: Supplementary file 1 [file MBO3-15-e70397-s001.docx]

**Supplementary Figure 1**. Flowchart of the dairy farm and dairy plant processing stages, outlining the sequence from raw milk collection to the production of cheese, yogurt, and cream-based products.

**:** represent sites where environmental or equipment surface samples were taken, : represent specific processing steps where product samples were collected.
