# Supplementary material for: Genetic Diversity and Epidemiological Overlap of Staphylococcus aureus at the Animal–Food–Environment–Human Interface Within a One Health Framework
Source: Microbiologyopen. 2026 Sep 3;15(5):e70397. doi: 10.1002/mbo3.70397 (PMC13542186; doi:10.1002/mbo3.70397)
Supplement: Supplementary file 2 [file MBO3-15-e70397-s003.docx]

**Genetic Diversity and Epidemiological Overlap of *Staphylococcus aureus* at the Animal–Food–Environment–Human Interface within a One Health Framework**

Pinar SAGIROGLU^a^, Dursun Alp GUNDOG^b^, Candan GUNGOR^c^, Kurşat KOSKEROGLU^d^, Mustafa Altay ATALAY^a^, Adalet DISHAN^b^, Yeliz UCAR^d^, Aytac AKCAY^e^, Huseyin Burak DISLI^f^, Harun HIZLISOY^d^, Mukaddes BAREL^d^, Tekin KECECI^d^, Nurhan ERTAS ONMAZ^d*^

^a^Department of Medical Microbiology, Faculty of Medicine, Erciyes University, Kayseri, Türkiye

^b^Veterinary Faculty, Department of Food Hygiene and Technology, Yozgat Bozok University, Yozgat, Türkiye

^c^Department of Veterinary Food Hygiene and Technology, Faculty of Veterinary Medicine, Necmettin Erbakan University, Ereğli, Türkiye

^d^Department of Veterinary Public Health, Faculty of Veterinary Medicine, Erciyes University, Kayseri, Türkiye

^e^Department of Biostatistics, Ankara University, Veterinary Faculty, Ankara, Türkiye

^f^Eville and Jones, Century House, 1275 Century Way, Thorpe Park, Leeds, UK

***Corresponding author:** Nurhan ERTAS ONMAZ E-mail: [nertas@erciyes.edu.tr](mailto:nertas@erciyes.edu.tr)

**Materials and Methods**

**Sample collection and study population**

A total of 560 samples were collected in Kayseri between August 2020 and March 2022, following a comprehensive farm-to-fork approach across distinct dairy and meat production systems within a One Health framework. The sampling framework was specifically designed to ensure full traceability, enabling the longitudinal monitoring of *S. aureus* across the entire production continuum. Facilities were selected based on permission granted to access all production stages, from primary animal handling to final product packaging.

For the dairy chain, two milk processing plants representing different production models were selected after permission was obtained from facility management. The first plant operated as a fully integrated enterprise with its own on-site dairy farm, enabling synchronized sampling of cattle, the milking environment, and derived dairy products. The second plant sourced raw milk from four small independent family farms (10–12 cows per farm) located in different areas of the same province, and sampling was coordinated to coincide with the arrival of raw milk at the processing plant. Sampling procedures were performed following established protocols described by Gundog et al. (2024). A total of 146 samples were collected along the dairy chain, including cattle nasal swabs (n=50), manger swabs (n=20), and farm worker samples (n=20) at the farm level; worker samples (n=14), equipment samples (n=10), environmental surface swabs (n=8), and raw tank milk samples (n=10) within the processing plants; and final dairy products, including cheese (n=5), yogurt (n=5), and cream (n=4). The dairy production workflow and corresponding sampling points are presented in Figure S1.

For the meat production system, two large-scale integrated enterprises were included after permission was obtained from facility management. Each enterprise comprised a livestock farm, slaughterhouse, and meat processing units, enabling systematic and traceable sampling from live animals to final processed products. Initially, primary surveillance was conducted at the farm level, and subsequent sampling stages were strictly synchronized with the scheduled slaughter dates of the same animal cohorts. To ensure full traceability, samples were collected from the same traced animal groups throughout slaughtering and processing operations. Sampling was performed as described by Gungor et al. (2021) and Koskeroğlu et al. (2026) and included cattle nasal swabs (n=50), environmental surface swabs (n=10), manger swabs (n=17), and worker nasal swabs (n=20) at the livestock farm; worker samples (n=30), carcass swabs (n=50), environmental surface swabs (n=50), and equipment samples (n=76) at the slaughterhouse; and finished meat products including pastırma (n=10), sausage (n=15), and salami (n=15), dried meat samples collected before edible paste coating during pastırma production (n=10), other semi-processed meat product samples collected during sausage and salami production (n=30), worker samples (n=10), environmental surface swabs (n=10), and equipment samples (n=11) from the meat processing plant, totaling 414 samples across the meat production continuum. The meat production workflow and corresponding sampling points throughout the farm, slaughterhouse, and processing stages are illustrated in Figure S2.

To provide a comparative One Health perspective, 50 clinical MRSA isolates were obtained from the culture collection of Erciyes University Faculty of Medicine. These isolates were recovered from diverse infection types, including wounds (n=22), abscesses (n=11), urinary tract infections (n=8), and blood samples (n=9).

The research protocol adhered to ethical guidelines and received formal approval from Erciyes University’s Human Research (2019/155) and Animal Ethics (19/036) Committees. Written informed consent was obtained voluntarily from all participating personnel. To minimize the influence of antimicrobial exposure, individuals and animals with antimicrobial treatment histories within the previous three months and one month, respectively, were excluded from sampling.

**Construction and Visualization of the spa-Based Minimum Spanning Tree**

To construct the Minimum Spanning Tree (MST), a custom Python script (version 3.10.2) was developed utilizing the NetworkX (v3.1) and Matplotlib libraries. The clustering was fundamentally based on the pairwise alignment of *spa* repeat sequences.

**Data Filtering and Preprocessing:** Prior to network construction, a programmatic preprocessing step was applied. Isolates designated as Non-typeable ("NT") were automatically filtered out from the dataset, as they lack the sequence data necessary for distance calculations. To ensure the visualization of the complete, unfiltered population structure and retain all potentially relevant genetic information, short *spa* types containing fewer than five repeat units were explicitly retained and integrated into the analysis.

**Distance Calculation:** The genetic distance between any two given *spa* types was calculated using the Levenshtein edit distance algorithm. This algorithm determines the minimum number of operations (insertions, deletions, or substitutions) required to transform one repeat sequence into another, quantitatively reflecting micro-evolutionary changes.

**MST Algorithm and Parameters:** A complete weighted undirected graph was initially generated, where nodes represented distinct *spa* genotypes and edges represented the calculated Levenshtein distance between them. The minimum_spanning_tree function from the NetworkX library was then applied using Kruskal’s algorithm to extract the MST, representing the subset of edges that connects all nodes with the minimum total edge weight.

**Visualization Parameters:** For the network layout, the Fruchterman-Reingold force-directed algorithm (spring_layout in NetworkX) was utilized. The spatial parameters were iteratively optimized (k=3.5, iterations=1000) to ensure an expansive and clear node distribution, preventing graphical overlap. Node areas were scaled proportionally to the square root of the isolate frequency to visually represent prevalence without dominating the graphical space. To maximize readability against the color-coded pie charts, a white path-effect stroke was applied to all node labels. Edge thicknesses and line styles were strictly categorized based on the exact Levenshtein distance to mathematically indicate the degree of similarity (e.g., width 3.5 solid lines for distance ≤ 2.2; width 2.0 solid lines for 2.2 < distance ≤ 3.4, etc.).

**Data Formatting:** The script utilizes the pandas library to parse the dataset. By default, the script is configured to read the data starting from the third row (header=2), assuming the first two rows contain preliminary metadata. The dataset must include the following exact column names (case-sensitive) for the algorithm to properly identify and process the parameters:

- spa-type: This column must contain the assigned *spa* genotype (e.g., t127, t005). Isolates that cannot be typed should be designated strictly as "NT".
- Spa Repeats: This column must contain the repeat succession sequence. The parsing function is dynamically designed to extract individual repeat units whether they are separated by hyphens, commas, or spaces (e.g., 07-23-12-34 or 07,23,12,34).
- Sample Source: This column must specify the epidemiological origin or host of the sample (e.g., Hospital, Dairy Farm, Slaughterhouse). The script dynamically aggregates these categorical values to calculate proportions and generate the color-coded pie charts within each node.

**Code Availability:** The complete Python script used for this analysis, along with detailed usage instructions, is provided in Supplementary File "MST_NetworkX_Code.txt" and is publicly available at the GitHub repository: <https://github.com/gundogalp-collab/Staph-MST-Analysis>.

 **Figure S1**. Flowchart of the dairy farm and dairy plant processing stages, outlining the sequence from raw milk collection to the production of cheese, yogurt, and cream-based products.

**:** represent sites where environmental or equipment surface samples were taken, : represent specific processing steps where product samples were collected.

**Figure S2.** Flowchart of the farm, slaughterhouse, and meat processing stages at the visited facility, illustrating the progression from live animal handling to the production of pastirma, sausage, and salami.

represent sites where environmental or equipment surface samples were taken, : represent specific processing steps where product samples were collected.

**References**

Gundog, D. A., Ozkaya, Y., Gungor, C., Ertas Onmaz, N., & Gonulalan, Z. (2024). Pathogenic potential of meat-borne coagulase negative staphylococci strains from slaughterhouse to fork. *International Microbiology 2024 27:6*, *27*(6), 1781–1793. https://doi.org/10.1007/S10123-024-00500-2

Gungor, C., Barel, M., Dishan, A., Burak Disli, H., Koskeroglu, K., & Onmaz, N. E. (2021). From cattle to pastirma: Contamination source of methicillin susceptible and resistant *Staphylococcus aureus* (MRSA) along the pastirma production chain. *LWT*, *151*, 112130. https://doi.org/10.1016/J.LWT.2021.112130

Koskeroglu, K., Onmaz, N. E., Gundog, D. A., Gungor, C., Gungor, G., Imre, K., & Morar, A. (2026). Tracking persistent and resistant Enterococcus faecalis and E. faecium from farm to fork: biofilm-linked risks in antibiotic resistance of isolates. *Veterinary Research Communications 2026 50:2*, *50*(2), 100-. https://doi.org/10.1007/S11259-025-11061-8
