# Supplementary material for: Genetic Diversity and Epidemiological Overlap of Staphylococcus aureus at the Animal–Food–Environment–Human Interface Within a One Health Framework
Source: Microbiologyopen. 2026 Sep 3;15(5):e70397. doi: 10.1002/mbo3.70397 (PMC13542186; doi:10.1002/mbo3.70397)
Supplement: Supplementary file 3 [file MBO3-15-e70397-s004.docx]

**Supplementary Figure 2.** Flowchart of the farm, slaughterhouse, and meat processing stages at the visited facility, illustrating the progression from live animal handling to the production of pastirma, sausage, and salami.

represent sites where environmental or equipment surface samples were taken, : represent specific processing steps where product samples were collected.
